# Specialist palliative care support is associated with improved pain relief at home during the last 3 months of life in patients with advanced disease: analysis of 5-year data from the national survey of bereaved people (VOICES)

**DOI:** 10.1186/s12916-019-1287-8

**Published:** 2019-03-22

**Authors:** Yousuf ElMokhallalati, Natalie Woodhouse, Tracey Farragher, Michael I. Bennett

**Affiliations:** 1 Academic Unit of Palliative Care, Leeds Institute of Health Sciences (LIHS), School of Medicine, University of Leeds, Room 10.39, Level 10, Worsley Building, Clarendon Way, Leeds, LS2 9NL UK; 2 Academic Unit of Public Health, Leeds Institute of Health Sciences, University of Leeds, Leeds, UK

**Keywords:** Palliative care, Pain management, Advance care planning, Home, Quality of Life, End-of-life care, Primary care

## Abstract

**Background:**

Studies have shown that more than half of patients with advanced progressive diseases approaching the end-of-life report pain and that pain relief for these patients is poorest at home compared to other care settings such as acute care facilities and hospice. Although home is the most common preferred place of death, the majority of deaths occur outside the home. Specialist palliative care is associated with improved quality of life, but systematic reviews of RCTs have failed to show a consistent association with better pain relief. The aim of this study was to examine the factors associated with good pain relief at home in the last 3 months of life for people with advanced progressive disease.

**Methods:**

Data were obtained from the National Bereavement Survey in England, a cross-sectional post-bereavement survey of a stratified random sample of 246,763 deaths which were registered in England from 2011 to 2015. From 110,311 completed surveys (45% response rate), the analysis was based on individual-level data from 43,509 decedents who were cared for at home before death.

**Results:**

Decedents who experienced good pain relief at home before death were significantly more likely to have received specialist palliative care (adjusted OR = 2.67; 95% CI, 2.62 to 2.72) and to have a recorded preferred place of death (adjusted OR = 1.87; 95% CI, 1.84 to 1.90) compared to those who did not. Good pain relief was more likely to be reported by a spouse or partner of the decedents compared to reports from their son or daughter (adjusted OR = 1.50, 95% CI, 1.47 to 1.53).

**Conclusion:**

This study indicates that patients at home who are approaching the end-of-life experience substantially better pain relief if they receive specialist palliative care and their preferred place of death is recorded regardless of their disease aetiology.

## Introduction

Pain is a highly prevalent and debilitating problem among people with advanced progressive disease [1, 2]. Studies have shown that more than 50% of patients with advanced cancer and non-cancer diseases reported pain, and the prevalence may increase as they approach the end of life [2–4]. Managing pain in people approaching the end of life is a major concern for health care professionals and a global public health priority [5, 6]. Despite increased availability of strong opioids, many patients still do not receive adequate analgesia for their pain [7]. Even in the UK, access to and duration of opioid treatment is limited for patients before they die, and people aged 60 years or older are less likely to receive opioids compared to younger patients [8, 9].

Numerous studies have consistently shown a mismatch between expressed preferences for place of death and actual place of death with most people preferring to die at home but the majority dying in hospital [10, 11]. Issues such as carer burden or difficulties in controlling pain and other symptoms at home make the majority of deaths occur outside the home [12, 13]. This is supported by evidence from the National Survey of Bereaved People (VOICES) in England which showed that pain relief is poorest for people who received end-of-life care at home compared to those dying in acute care facilities or hospice [14]. Only 19% of respondents reported pain to be completely relieved in people who were cared at their own home in comparison with 64% in hospices, 43% in care homes and 40% in hospital [15]. UK policy supports the need for improved quality of care for people dying at home, particularly relief from pain [16–18].

Although specialist palliative care (compared to usual care) is associated with improved quality of life, there is inconclusive evidence from meta-analyses about the effect on reducing pain and symptom burden [19–25]. Pain relief in older adults and those with non-cancer disease may be harder to obtain because these patients have limited access to specialist palliative care services compared to younger patients and those diagnosed with cancer [14, 26–29]. We aimed to examine the factors associated with good pain relief at home in the last 3 months of life for people with advanced progressive illness. Specifically, we sought to examine the relationship between the extent of pain relief at home and receiving specialist palliative care.

## Methods

### Population and data source

The National Survey of Bereaved People (VOICES, Views of Informal Carers - Evaluation of Services) is a nationally representative cross-sectional survey which was conducted in England annually for 5 years, 2011–2015, to collect information about the quality of end-of-life care, particularly in the last 3 months of life [30]. The survey was commissioned by NHS England and administered by the Office for National Statistics (ONS). The survey’s results are based on a relative’s or friends’ perspective on the quality of end-of-life care provided to the decedent. Previous analyses of VOICES data have been used to inform national policy on end-of-life care service and assess and evaluate the quality of end-of-life care in different settings (home, hospital, care homes and hospices) [31]. Every year, a stratified sample of around 49,000 adults was selected from deaths which are registered in England. The VOICES questionnaire was sent by post to the person who registered the death of the decedent who is normally a family member or a close friend. Respondents were contacted once between 4 and 11 months after death (two further reminder questionnaires were sent if there was no response). The sampling weight and non-response weight were created by the ONS for each year. The sampling weight and non-response weight were then combined by taking the product of the two. We used the combined weight to adjust for sampling and non-response biases. Further information on VOICES methodology is available from the ONS [30].

### Sampling

We obtained data from five annual VOICES surveys conducted between 2011 and 2015. During this period, 246,763 people were invited to participate, of whom 110,311 (45%) returned a completed questionnaire. Because VOICES survey does not contain information about palliative care in settings other than home, we examined factors associated with good pain relief at home in the last 3 months of life for people with advanced progressive illness. In addition, the main outcome was the success of pain relief at home which was applied only to decedents who had pain at home in the last 3 months of life.

The following exclusion criteria were applied:

1. Decedent who died suddenly or were not ill prior to death.
2. Decedent who did not spend any time at home in the last 3 months of life.
3. Decedent who did not have any pain at home in the last 3 months of life.

### Independent variables

Respondent characteristics included age, gender and relation to the decedent (spouse/partner, son/daughter, other). Decedent characteristics included age, gender, cause of death (cancer or non-cancer), index of multiple deprivation (IMD) quintiles (1 = most deprived, 5 = least deprived), duration of illness before decedent died.

We developed variables for service characteristics labelled receiving specialist palliative care at home in the last 3 months of life (yes or no), recorded preference for place of death (yes or no) and urgent care provided out of hours (once or twice, three times or more, not at all).

#### Specialist palliative care proxy measure

Respondents were asked about the decedent, ‘when he/she was home in the last three months of life, did he/she get any help from any of the services: hospice home care nurse or specialist, hospice at home service, Macmillan nurse or Marie Curie nurse? (Macmillan and Marie Curie are UK-based charities that fund clinical nurse specialists in palliative care who deal with cancer and non-cancer patients). If respondents answered ‘yes’ to any of the three questions, it was assumed that their relative had received specialist palliative care in the last 3 months of their life, defined as professionals or services whose core activity is providing palliative care. If responders answered ‘no’ to all three questions, it was considered for the purposes of this research that their relative had not received specialist palliative care in the last 3 months of their life.

#### Recorded preference for place of death

The survey asks about the decedent ‘Did she/he ever say where she/he would like to die?’ If responders answered ‘yes’, they were asked about the preferred place of death. After that, they were asked ‘Did the health care staff have a record of this?’ Having a preference recorded for place of death in the medical records was used as a positive indicator for this variable.

### Outcomes

The primary outcome was the extent of pain relief at home. We collapsed the response categories included in the survey for ease of interpretation into: (1) good pain relief (pain relieved completely, all of the time and completely, some of the time) and (2) poor pain relief (pain relieved partially, not at all).

### Statistical analysis

Based on individual-level survey data, numbers and percentages (both unweighted and weighted) were calculated to summarise decedent and respondent characteristics. Logistic regression models were used to examine the association between decedent and respondent characteristics and good pain relief. All variables that had a *p* value less than 0.1 univariately (to account for potential collinearity) were included in an initial multivariable model. As the aim of the analysis was to identify factors associated with good pain relief, rather than develop the most parsimonious model, variables were retained in the final multivariable model if they improved the fit of the model based on the likelihood-ratio test (using backward selection *p* <  0.05). In the final multivariable logistic regression model, we assessed potential multicollinearity using the variance inflation factor (VIF). Statistical software IBM SPSS statistics version 24 was used for data management and analysis. We used the published weights for the VOICES survey in the analysis to account for the study design and to adjust for non-response bias [30].

## Results

**Fig. 1:**
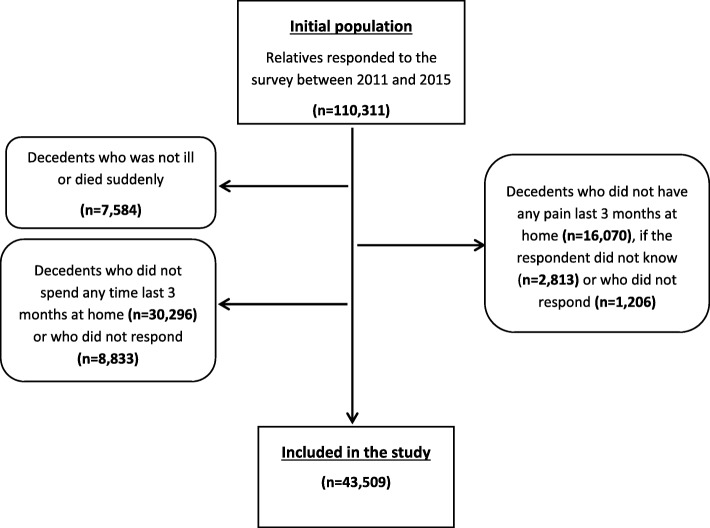
Flow chart showing records included in this study as well as reasons for exclusion

Of the 110,311 respondents to the VOICES questionnaire from 2011 to 2015, 66,802 (60.6%) respondents did not meet the inclusion criteria and were excluded (Fig. 1). Therefore, 43,509 (39.3%) of respondents were included in our analysis. Around 51.6% of decedents were female and 59.8% of survey respondents were female (Table 1). Decedents aged 75 and older account for 60.9% of the study population. Over half of decedents (52.1%) were ill for more than a year prior to death, and 51.2% of deaths were from non-cancer disease. Data were missing for the following variables: How long had she or he been ill prior to death (484, 1.1%), respondent age (531, 1.2%), respondent’s relationship to decedent (733, 1.6%) and respondent sex (2298, 5.1%).

**Table 1:** Decedent and respondent characteristics

	Number of respondents	Unweighted % [95%% CI]	Weighted % [95%% CI]
Decedent’s age			
18–74	14,888	34.2 (33.8–34.7)	39.1 (39.0–39.3)
75–84	13,903	32.0 (31.5–32.4)	30.9 (30.7–31.0)
85+	14,718	33.8 (33.4–34.3)	30.0 (29.8–30.1)
Decedent’s gender			
Male	20,820	47.9 (47.4–48.3)	48.4 (48.2–48.6)
Female	22,689	52.1 (51.7–52.6)	51.6 (51.5–51.8)
Cause of death			
Cancer	21,737	50.0 (49.5–50.4)	48.8 (48.6–48.9)
Non-cancer	21,772	50.0 (49.6–50.5)	51.2 (51.1–51.4)
Deprivation (IMD)			
1 (most deprived)	7197	16.5 (16.2–16.9)	18.6 (18.5–18.8)
2	8191	18.8 (18.5–19.2)	19.8 (19.7–20.0)
3	9476	21.8 (21.4–22.2)	21.5 (21.4–21.7)
4	9458	21.8 (21.4–22.2)	20.7 (20.5–20.8)
5 (least deprived)	9187	21.1 (20.7–21.5)	19.4 (19.3–19.5)
Length of illness prior to death			
< 1 month	4607	10.7 (10.4–11.0)	10.5 (10.4–10.4)
> 1 month, < 6 month	9745	22.6 (22.2–23.0)	22.0 (21.9–22.1)
> 6 month, < 1 year	6631	15.4 (15.1–15.7)	15.4 (15.2–15.5)
> 1 year	22,106	51.3 (50.8–51.8)	52.1 (52.0–52.3)
Respondent’s age			
18–49	7663	17.9 (17.5–18.2)	19.6 (19.5–19.7)
50–59	12,531	29.1 (28.7–29.6)	29.1 (29.0–29.3)
60–69	13,772	31.2 (30.8–31.7)	30.5 (30.4–30.7)
70–79	6594	15.3 (15.0–15.7)	14.8 (14.7–14.9)
80+	2952	06.5 (06.3–06.8)	06.0 (05.9–06.1)
Respondent’s gender			
Male	16,632	40.3 (39.8–40.7)	40.3 (40.2–40.5)
Female	24,690	59.7 (59.3–60.2)	59.7 (59.5–59.8)
Respondent’s relationship to decedents			
Wife/husband/partner	14,670	34.2 (33.8–34.7)	35.5 (35.4–35.7)
Son/daughter	22,745	53.1 (52.6–53.6)	50.7 (50.5–50.9)
Other	5420	12.7 (12.3–13.0)	13.8 (13.6–13.9)

Care and service characteristics by cause of death are shown in Table 2. Overall, 35.7% of decedents received specialist palliative care at home and 24.6% had a recorded preference for place of death. About 78.7% of decedents had at least one out-of-hours service contact in the last 3 months of life. Cancer decedents were more likely to receive specialist palliative care support at home (66.2% vs 9.9%), have a recorded preference of place of death (36.6% vs 13.1%) and to contact out-of-hours service in the last 3 months of life (81.6% vs 75.9%) compared to non-cancer decedents.

**Table 2:** Care and service characteristics in the last 3 months by cause of death

	Total % [95%% CI]	Cancer % [95%% CI]	Non-cancer % [95%% CI]
Specialist palliative care at home (yes)	35.7 (35.5–35.8)	62.7 (62.5–63.0)	9.9 (9.7–10.0)
Recorded preference for place of death (yes)	24.6 (24.5–24.7)	36.6 (36.4–36.9)	13.1 (12.0–13.3)
Urgent care provided out of hours			
Not at all	21.3 (21.2–21.4)	18.4 (18.2–18.6)	24.1 (23.9–24.3)
Yes	78.7 (78.6–78.8)	81.6 (81.4–81.8)	75.9 (75.7–76.1)
Once or twice	33.1 (33.0–33.3)	35.9 (35.7–36.1)	30.4 (30.2–30.7)
Three or more	45.6 (45.4–45.8)	45.7 (45.5–45.9)	45.5 (45.2–45.7)

All percentages were weighted by sampling weight and non-response weight

Pain relief by cause of death is presented in Table 3. Overall, just under half of the decedents in our study experienced good pain relief (47.8%). For decedents with non-cancer disease, around 39.6% had good pain relief compared to 56.4% of cancer decedents (*p* <  0.001).

**Table 3:** Proportions of decedents with good pain relief in the last 3 months by cause of death and service characteristics

	Total % [95%% CI]	Cancer % [95%% CI]	Non-cancer % [95%% CI]
Overall	47.8 (47.6–47.9)	56.4 (56.1–56.6)	39.6 (39.3–39.8)
Specialist palliative care at home			
Yes	66.2 (65.9–66.5)	66.7 (66.4–67.0)	62.9 (62.2–63.7)
No	37.5 (37.3–37.7)	38.9 (38.5–39.2)	37.3 (36.8–37.3)
Recorded preference for place of death			
Yes	66.0 (65.7–66.4)	69.0 (68.6–69.4)	58.1 (57.5–58.8)
No	41.7 (41.5–41.9)	49.0 (48.7–49.3)	36.6 (36.4–36.9)
Urgent care provided out of hours			
Not at all	45.3 (44.9–45.7)	54.9 (54.4–55.5)	38.2 (37.8–38.7)
Once or twice	48.6 (48.3–48.9)	56.3 (55.9–56.7)	39.9 (39.5–40.3)
Three or more	49.6 (49.3–49.8)	58.2 (57.9–58.6)	41.2 (40.9–41.6)

All percentages were weighted by sampling weight and non-response weight

**Fig. 2:**
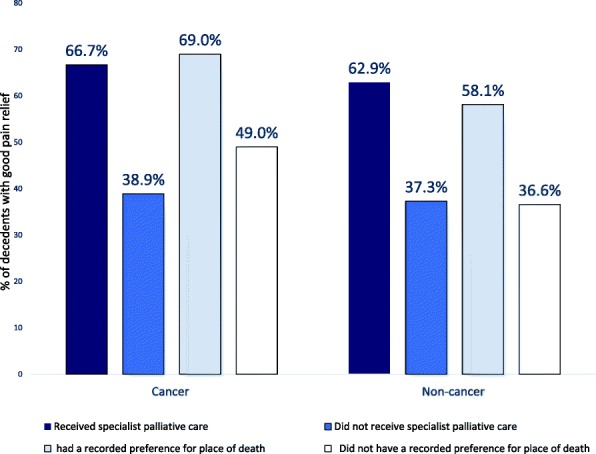
Proportions of decedents with good pain relief at home by cause of death and palliative care status during last 3 months of life

Table 3 also compares pain relief by service characteristics. This shows that 66.2% of decedents who received specialist palliative care services at home experienced good pain relief compared to 37.5% of those who did not (*p* <  0.001). Among patients who had a recorded preference for place of death, 66.0% had good pain relief in comparison with only 41.7% of decedents who did not (*p* <  0.001). Good pain relief stratified by cause of death and service characteristics is illustrated in Fig. 2.

Univariately all characteristics were significantly associated (*p* <  0.001) with good pain relief at home (Table 4) and remained in the final multivariable model. The multivariable model revealed a significantly higher chance of experiencing good pain relief among those who received specialist palliative care at home (adjusted OR = 2.67; 95% CI, 2.62 to 2.72) and who had a recorded preference for place of death (adjusted OR = 1.87; 95% CI, 1.84 to 1.90) in comparison with those who did not, keeping all other characteristics constant (Table 4). In addition, compared to decedents who did not contact out-of-hours services, decedents who contacted out-of-hours services three times or more were more likely to have good pain relief (adjusted OR = 1.05; 95% CI, 1.03 to 1.07), while decedents with one or two out-of-hours service contacts experienced worse pain relief (adjusted OR = 0.89; 95% CI, 0.87 to 0.91).

**Table 4:** Logistic regression of factors associated with good pain relief at home

	Univariate model			Multivariable model		
	OR	95% CI	*p* value	OR	95% CI	*p* value
Specialist palliative care at home						
No^b^	1	–		1	–	–
Yes	3.26	3.21 to 3.31	< 0.001	2.67	2.62 to 2.72	< 0.001
Recorded preference for place of death						
No^b^	1			1		
Yes	2.72	2.68 to 2.77	< 0.001	1.87	1.84 to 1.90	< 0.001
Urgent care provided out of hours			< 0.001^a^			< 0.001^a^
Not at all^b^	1	–		1	–	–
Once or twice	1.14	1.12 to 1.17	< 0.001	0.89	0.87 to 0.91	< 0.001
Three times or more	1.19	1.17 to 1.21	< 0.001	1.05	1.03 to 1.07	< 0.001
Cause of death						
Non-cancer^b^	1	–		1	–	–
Cancer	1.97	1.95 to 2.00	< 0.001	1.08	1.06 to 1.10	< 0.001
Decedent’s age			< 0.001^a^			< 0.001^a^
18–74^b^	1	–		1	–	–
75–84	0.86	0.84 to 0.87	< 0.001	1.22	1.19 to 1.24	< 0.001
85+	0.78	0.77 to 0.80	< 0.001	1.46	1.43 to 1.50	< 0.001
Decedent’s gender						
Male^b^	1	–		1	–	–
Female	0.92	0.90 to 0.93	< 0.001	1.03	1.01 to 1.04	0.001
Deprivation (IMD)			< 0.001^a^			< 0.001^a^
1 (most deprived)^b^	1	–		1	–	–
2	1.18	1.15 to 1.20	< 0.001	0.98	0.96 to 1.01	0.16
3	1.07	1.05 to 1.09	< 0.001	1.03	1.01 to 1.05	0.04
4	1.14	1.12 to 1.17	< 0.001	1.04	1.01 to 1.06	0.003
5 (least deprived)	1.17	1.14 to 1.19	< 0.001	1.01	0.98 to 1.03	0.54
Respondent’s relationship to decedents			< 0.001^a^			< 0.001^a^
Son/daughter^b^	1	–		1	–	–
Wife/husband/partner	1.68	1.66 to 1.71	< 0.001	1.50	1.47 to 1.53	< 0.001
Other	1.05	1.03 to 1.07	< 0.001	1.13	1.10 to 1.16	< 0.001
Length of illness prior to death.			< 0.001^a^			< 0.001^a^
> 1 year^b^	1	–		1	–	–
< 1 month	0.60	0.59 to 0.62	< 0.001	0.92	0.90 to 0.95	< 0.001
> 1 month, < 6 month	0.86	0.84 to 0.87	< 0.001	0.91	0.89 to0.92	< 0.001
> 6 month, < 1 year	1.10	1.08 to 1.12	< 0.001	1.01	0.98 to1.03	0.63

*OR* odds ratio, *CI* confidence interval, *IMD* Index of Multiple Deprivation

^a^*P* value for overall effect

^b^Used as a reference variable

The results also showed that women had 3% higher odds of good pain relief than compared to men (adjusted OR = 1.03; 95% CI, 1.01 to 1.04), keeping all other characteristics constant. In addition, shorter duration of illness was associated with reduced probability of experiencing good pain relief in comparison with decedents with illness for more than a year; that is, the odds of good pain relief were 9% lower for decedents who were ill between 1 month and 6 months prior to death in comparison with decedents with illness for more than a year. Moreover, decedents who died of cancer were more likely to experience good pain relief (adjusted OR = 1.08; 95% CI, 1.06 to 1.10), compared to non-cancer decedents and keeping all other characteristics constant.

Decedents aged 75 and older were more likely to experience good pain relief, compared to younger decedents. For example, the odds of experiencing good pain relief were 46% higher among decedents aged 85 and older compared to younger decedents aged 74 or younger. Deprivation (IMD) was significantly associated with good pain relief, but in a nonlinear pattern. For example, compared to the most deprived quintile, decedents who lived in the second least deprived quintile had significantly higher odds of good pain relief while those in the least deprived quintile had no difference in their odds of good pain relief. The respondent’s relationship with decedents was also significantly associated with good pain relief. Good pain relief was more likely to be reported by a spouse or partner of the decedent compared to a son or daughter of the decedent (adjusted OR = 1.50, 95% CI, 1.47 to 1.53), keeping all other characteristics constant.

Multicollinearity did not appear to be present, based on an evaluation using the variance inflation factor (VIF). All VIF scores are less than 1.7. These scores are well below the cutoff values of between 5 and 10, in which collinearity may be problematic [32].

## Discussion

Our analysis of 43,509 patients who were cared for at home before death showed that receiving specialist palliative care and have a recorded preference for place of death were found to be strongly and independently associated with good pain relief in the last 3 months of life. These findings have contributed to evidence supporting the need for, and the benefits of, specialist palliative care and recording preferences for place of death for patients with advanced disease. Furthermore, we have demonstrated that respondents who were spouses or partners of the decedents were much more likely to report better pain relief that respondents who were sons or daughters.

A major strength of this study is that we used data from the first national survey on the quality of end-of-life care in England. The response rate was modest (45%) which could lead to bias. However, the response rate provides a suitable sample size for analysis at the national level and the weighting method we used corrected for non-response and sample biases [30]. We have also combined 5 data sets, 2011–2015 to increase the sample size in order to improve data robustness. The validated questionnaire and the large sample size provided good statistical power from which generalisable findings can be made.

Our study has a number of limitations. A key drawback is that it was an analysis of a post-bereavement survey which used the perceptions of decedent’s relatives as proxy measure of the quality of care experienced by the patient. Retrospective ratings of end-of-life care by decedent’s relatives could be different from the actual experience of the decedent. Research is difficult to conduct on patients with advanced progressive disease and although the validity and reliability of proxies are questionable, it may need to be accepted and utilised as a valuable part of end-of-life research [33]. Without this proxy measure, there are only few, poorly powered studies to inform important policy documents and the practice of end-of-life care. Moreover, retrospective studies allow for more representative samples to be studied as they are not limited to diseases such as cancer that have an identifiable terminal phase. It is better to have imperfect recollections from proxies than it is to have no perceptions of end-of-life care at all [33]. In addition, recall bias cannot be excluded due to the timing of data collection being 4–11 months after death and the possibility of inaccurately recalling subjective aspects such as pain.

Another important limitation of the findings is that we were only able to demonstrate an association between improved pain relief and specialist palliative care and recorded preference for place of death, but we cannot demonstrate causality. However, it is entirely plausible that contact with specialist palliative care or discussing and recording preferred place of death results in better pain relief, particularly because of the emphasis by specialist palliative care clinicians on symptom management and expertise in use of opioid analgesia. The counter-argument that patients with good pain relief ‘cause’ a referral to specialist palliative care is much less plausible as eligibility criteria for referral to specialist palliative care services include symptoms that are uncontrolled or complicated, i.e. patients have poor pain relief not good pain relief [34]. This might also imply that patients referred to specialist palliative care had worse baseline pain than those not referred and that the impact of specialist palliative care on pain relief is underestimated in this analysis. The association between good pain relief and recording of preferred place of death is more difficult to explain. It may represent engagement in broader aspects of advance care planning which includes pain management strategies, and perhaps reflects greater communication and engagement with healthcare professionals, both of which might lead to better pain outcomes. The type of respondent influenced recollection of pain relief; spouses and partners rated pain relief better than sons or daughters. Spouses and partners are likely to have had closer contact than sons or daughters and perhaps represent a more reliable account. Conversely, spouses and partners are more likely to be older and have potentially different (lower) expectations of healthcare services and interventions than younger sons and daughters resulting in inflated views of outcomes. Finally, we cannot exclude the possibility of uncontrolled confounding by factors that were not measured within the survey, for example the presence of other symptoms that might trigger palliative care referral, other services that patients received that were not captured within the survey questions or presence of depression or anxiety within decedents or respondents. Interestingly, decedents aged 75 and older were less likely to experience good pain relief, compared to younger decedents in the univariable analysis, but following adjustment and multivariable analysis, decedents aged 75 and older had a significantly higher odds of good pain relief compared to younger decedents (Table 4). The reason for this swing in direction of the association might be due to confounding with other variables in the final multivariable model. While we checked and found no evidence of a high degree of multicollinearity, associations between both the outcome and age and another variable(s) are likely to be present.

This is the largest study that shows a significant association between receiving specialist palliative care at home and improved pain relief within routine clinical services. Previous research has found an inconclusive relationship between palliative care support and improved pain relief. A US national study by Teno et al., which is one of very few national surveys of family perspectives on end-of-life care, supports our findings [35]. These authors found that in 512 patients who died at home, pain relief was significantly better in the 256 patients who received care at home with hospice services compared to receiving only home nursing services. In a systematic review and meta-analysis of trials [19], which examined the effectiveness of home palliative care, only 3 of 9 studies in which pain was an outcome measure found statistically significant positive effects on pain relief.

## Conclusion

Our study indicates that patients at home who are approaching the end-of-life experience substantially better pain relief if they receive specialist palliative care or have a recorded preference for place of death, regardless of their disease aetiology. Policymakers should consider how to ensure improvements in pain management for patients at home through advice and support from community specialist palliative care services.

## Abbreviations

CI: Confidence interval
IMD: Index of Multiple Deprivation
NHS: National Health Service
ONS: Office for National Statistics
OR: Odds ratio
RCTs: Randomised control trials
VIF: Variance inflation factor
VOICES: Views of Informal Carers - Evaluation of Services
